# Editorial: Women in brain imaging and stimulation in the era of artificial intelligence

**DOI:** 10.3389/fnhum.2026.1904700

**Published:** 2026-07-02

**Authors:** Wen Li, Ruogu Fang

**Affiliations:** 1 Department of Psychiatry and Behavioral Sciences, The University of Texas Health Science Center at Houston, TX, United States; 2 Department of Biomedical Engineering, University of Florida, Gainesville, FL, United States

**Keywords:** artificial intelligence, brain imaging, brain stimulation, neuroimaging, neuromodulation, women in neuroscience, EEG

The Brain Imaging and Stimulation section of Frontiers in Human Neuroscience launched this Research Topic series in 2022 with a straightforward premise: women investigators were leading consequential science in this field, and that work deserved a dedicated platform. Two volumes later (Fang et al., 2023; Hermiller et al., 2025), the premise has been borne out. What has changed is the landscape in which that science now unfolds. Artificial intelligence has moved from a set of promising techniques at the margins of neuroimaging into a central methodological force, reshaping how data are acquired, processed, and translated into clinical decisions. This third volume, “*Women in brain imaging and stimulation in the era of artificial intelligence*,” reflects that shift directly. Several contributions deploy AI as a primary engine of discovery or clinical innovation; others develop the experimental and biological foundations on which AI-assisted work will depend. Together they define what the intersection of inclusive neuroscience and AI-era methodology looks like in practice.

## Contributions of the present volume

The most explicit engagement with artificial intelligence in this Research Topic comes from Khan et al., whose deep learning framework MAGIC (Multitask Automated Generation of Intermodal CT perfusion maps) addresses one of the most time-sensitive challenges in emergency neurology. Stroke triage depends on CT perfusion imaging, yet the standard protocol requires intravenous contrast agents that are contraindicated in a substantial minority of patients, such as those with renal insufficiency, prior allergic reactions, or limited access to contrast-capable facilities. MAGIC pairs a generative adversarial network with physiology-informed loss terms to synthesize diagnostic-quality perfusion maps directly from non-contrast CT. Validated in a double-blinded study with seven neuroradiologists and vascular neurologists, the framework achieved performance comparable to conventional contrast-enhanced perfusion, an outcome with immediate implications for equitable stroke care. This work exemplifies a principle that runs through all three volumes of this series: that methodological innovation and equity are not separate agendas.

Chen et al. contribute a synthesis that provides essential conceptual scaffolding for the next generation of AI-assisted EEG diagnostics in neurodegeneration. Their review maps the relationship between oscillatory pathology, which is characterized by disrupted theta, alpha, and gamma dynamics, and default mode network dysfunction in Alzheimer's disease. The convergence of these two systems represents one of the most tractable targets for non-invasive, low-cost early detection. As machine learning tools for EEG interpretation mature, the mechanistic grounding offered by this review will be indispensable: AI-identified biomarkers that cannot be anchored to known neural circuit dysfunction remain difficult to validate and harder still to translate. Prior volumes in this series similarly emphasized the importance of building rigorous biological frameworks before deploying computational tools rather than afterward, a disciplinary ethic this work embodies.

Eng et al. bring AI-era logic to the clinic in a different register, presenting a proof-of-concept study of personalized non-invasive neuromodulation for obsessive-compulsive disorder. The intervention targets sensory-based urge states in OCD using stimulation parameters individualized to each patient's neural phenotype. This is a paradigm that depends on the kind of real-time neural decoding and closed-loop feedback that AI is increasingly enabling. The study demonstrates feasibility and preliminary efficacy, and it illustrates how the precision psychiatry framework that was largely theoretical in Volume I (Fang et al., 2023) and gaining methodological traction by Volume II (Hermiller et al., 2025) is now producing patient-level results.

Arrington et al. report methodology and preliminary findings from a combined accelerated theta burst stimulation (aTBS) and structured reading instruction protocol for persistent developmental dyslexia. By systematically integrating repetitive TMS-based neuroplasticity induction with evidence-based language intervention, the authors operationalize a closed-loop logic in which stimulation potentiates the neural substrate for skill acquisition. The methodological documentation is itself a contribution, which is detailed enough to support multi-site replication and, ultimately, the larger randomized trials that would justify AI-assisted participant selection and outcome prediction.

Jun et al. present a case-control neuroimaging investigation of adults with chronic Zika virus infection, a population whose long-term central nervous system sequelae remain poorly characterized. Conducted in a Caribbean cohort, the study documents structural and functional brain differences in chronically infected adults, shifting the literature's focus from congenital to acquired infection and from acute to chronic phase. This work opens a new neuroepidemiological line of inquiry with direct relevance for AI-enabled population-scale imaging surveillance in settings where resources for conventional clinical workup are constrained. The global health scope here is consistent with the series' commitment to research that travels beyond high-resource imaging environments.

Finally, Huang et al. examine how perceived class mobility shapes pain empathy in wealthy individuals from collectivist cultures, using event-related potentials to track the neural timing of social-affective processing. The finding that mobility beliefs modulate N2 and late positive potential components extends social neuroscience into underexplored intersections of class, culture, and empathic cognition. As AI tools for decoding complex ERP waveforms become more widely adopted, studies with this degree of experimental and cultural specificity become important validation anchors. This reminds researchers that learned ERP features require diverse, ecologically grounded training sets to generalize.

## Artificial intelligence: from tool to research context

The decision to subtitle this volume “in the Era of Artificial Intelligence” was not merely descriptive. It was a recognition that AI now shapes not only what questions are answerable but which questions get asked, which datasets get built, and whose brains end up represented in the models that clinical tools will eventually deploy. Research programs led by women investigators, including those showcased across all three volumes of this series, are disproportionately attentive to underrepresented populations, ecological validity, and methodological transparency. These are precisely the properties that determine whether an AI-augmented neuroimaging tool generalizes beyond the laboratory. The contributions here, taken together, argue that inclusive science and rigorous AI development are not parallel tracks but the same track.

At the same time, AI offers genuine opportunities to extend the scientific agenda this series has championed since its inaugural Research Topic. Automated quality control, large-scale data harmonization, and high-dimensional biomarker discovery all hold particular promise for research programs that have historically been constrained by limited sample sizes or single-site acquisition. MAGIC's contrast-free perfusion synthesis (Khan et al.) and the EEG-DMN framework proposed by Chen et al., each suggest how AI, when built on principled biological foundations, can expand access to capabilities previously reserved for well-resourced clinical centers. The series will continue to track this evolution.
